# Abundance of molecular triple ionization by double Auger decay

**DOI:** 10.1038/s41598-018-34807-8

**Published:** 2018-11-06

**Authors:** A. Hult Roos, J. H. D. Eland, J. Andersson, R. J. Squibb, D. Koulentianos, O. Talaee, R. Feifel

**Affiliations:** 1 Department of Physics, University of Gothenburg, Origovägen 6B, 412 58 Gothenburg, Sweden; 3 Department of Chemistry, Physical and Theoretical Chemistry Laboratory, Oxford University, South Parks Road, Oxford, OX1 3QZ United Kingdom; 4 Sorbonne Université, CNRS, Laboratoire de Chimie Physique-Matière et Rayonnement, F-75005 Paris Cedex 05, France; 2 Nano and Molecular Systems Research Unit, University of Oulu, P.O. Box 3000, University of Oulu, FI-90014 Finland

## Abstract

Systematic measurements of electron emission following formation of single 1s or 2p core holes in molecules with C, O, F, Si, S and Cl atoms show that overall triple ionization can make up as much as 20% of the decay. The proportion of triple ionization is observed to follow a linear trend correlated to the number of available valence electrons on the atom bearing the initial core hole and on closest neighbouring atoms, where the interatomic distance is assumed to play a large role. The amounts of triple ionization (double Auger decay) after 1s or 2p core hole formation follow the same linear trend, which indicates that the hole identity is not a crucial determining factor in the number of electrons emitted. The observed linear trend for the percentage of double Auger decay follows a predictive line equation of the form DA = 0.415 · N_ve_ + 5.46.

## Introduction

Formation of a core vacancy in an atom or molecule, upon photon absorption or high energy particle impact, will lead to relaxation of the system where one of the possible consequences is non-radiative secondary electron emission commonly denoted as Auger decay. The major non-radiative de-excitation process is a two-electron transition, in which one electron fills the core vacancy while another electron is ejected, leaving a doubly charged ion (dication) and one more electron in the continuum in addition to the photoelectron. Spectra of dications from this single Auger (SA) decay in polyatomic molecules are more complex than the spectra of isolated atoms, because molecular vibrations and dissociations come into play, and the increased number of valence electrons also implies an increased number of possible final double-hole states. These factors typically result in broad spectral profiles, where the intensity represents a convolution of contributions from many possible transitions. Double Auger (DA) decay is a three-electron transition in which two electrons are ejected upon the filling of the core vacancy, and is always less abundant than single Auger decay, but is also even more complex, as the number of possible final electronic states is even greater in the triply ionized product.

The existence of double Auger decay is well known experimentally and can be interpreted theoretically for atomic systems^1,2^. Our knowledge of the relative extent of double Auger decay is still very limited, and it is restricted mainly to atoms and very recently to simple molecules^3^. Many earlier works involving double ionization by core-vacancy decay^4–8^ have ignored the existence of double Auger decay as a competing ionization pathway, on the basis that its relative intensity is negligible. Some earlier work from this laboratory^9–11^ suggested, however, that the proportion of triple ionization by double Auger decay may be significant in some cases, and our previous investigation^3^ confirmed that it can reach 10% even in relatively simple carbon, nitrogen, and oxygen compounds.

To build further on that preliminary work^3^, we here present a systematic investigation covering a larger number of carbon, oxygen, fluorine, silicon, sulphur and chlorine compounds. The present experimental investigation is based on the combination of synchrotron radiation and a magnetic bottle time-of-flight spectrometer which is sensitive to multiple electron emission events at high electron collection and detection efficiency. The central question which we consider here is whether the percentage of DA decay varies as the core hole is created on different atomic sites or the same type of atom but in different molecular environments of complex molecular systems. Specifically, we investigate the Auger decay upon the formation of a carbon, oxygen, or fluorine 1s core hole, and upon the creation of a sulphur, chlorine, or silicon 2p core hole in a series of compounds.

## Results and Discussion

### Electron spectra from the different processes

Double and triple ionization spectra from single Auger and double Auger decay of the samples studied in the present work have some common characteristics which are important for the determination of the proportion of double Auger decay in these molecules after core hole formation. The first notable characteristic is that the energy distributions of the double Auger electrons are strongly asymmetric, with one fast and one slow electron. This is consistent with the theory on double Auger decay in atoms reported in refs^1,2^, and with our previous study^3^ on the amount of double Auger decay in simple molecules. Another characteristic is that the SA and DA spectra overlap in energy, i.e. there are some doubly ionized states above the threshold for triply ionized states. A trivial reason for this spectral overlap, besides resolution limitations, is that even in our magnetic bottle apparatus only about half of all emitted electrons are detected. As a result some real triple ionization events are detected as double ionization and appear in the energy range characteristic of double ionization. Subtraction of these false electron pairs requires knowledge of the actual detection efficiency. Overlap of SA and DA spectra also arises naturally because there are many ways that three electrons can be released from the orbitals of a molecule, producing excited triply charged states. Each triply-charged state will have Rydberg-like dicationic states converging towards it^12,13^. Many of these states can decay by dissociation to doubly charged fragments as a result of Coulomb repulsion instead of, or in competition with, autoionization to ground-state triply charged products.

A full state-by-state characterization of stepwise double Auger decay after core ionization in the molecules in this study is effectively impossible, because of the inherent lack of spectral resolution. Molecules in most dicationic and all tricationic states, including possible intermediate states, are highly dissociative, so vertical transitions to them give rise to broad overlapping bands in the spectra. Molecules, just like atoms will undergo indirect double Auger decay through intermediate dication states, which we cannot distinguish from a direct process, so in this work we concentrate on the final outcome, the fraction of triple ionization after core hole formation. This is the quantity that is of primary interest in the present context and is the ratio that should borne in mind when interpreting experiments where, for example, ion pairs, but not triples, are detected after core-hole formation.

### Fractions of double and triple ionization

The percentage of triple ionization upon creation of a single hole in the C 1s, O 1s, F 1s, Si 2p, S 2p, or Cl 2p orbital is looked for in each sample in coincidence with the corresponding photoelectron. The true fraction of triple ionization is obtained from the relative numbers of electron triples and pairs, corrected for the relevant efficiency of collection and detection at each energy; hereafter this composite quantity is simply termed collection efficiency to avoid needless repetition. As shown in our previous paper^3^, the correction factor is given by the expression ${f}_{{\rm{e}}}^{{\rm{SA}}}/({f}_{{\rm{e}}}^{\mathrm{DA},\mathrm{slow}}\cdot {f}_{{\rm{e}}}^{\mathrm{DA},\mathrm{fast}})$, where ${f}_{{\rm{e}}}^{{\rm{SA}}}$ is the efficiency for the single SA electron and ${f}_{{\rm{e}}}^{\mathrm{DA},\mathrm{slow}/\mathrm{fast}}$ are collection efficiencies for the slow and fast DA electrons. The collection efficiency for the photoelectrons is common to all subsequent decay events. Because of the asymmetric energy distribution for the DA electron pair, the expression can be simplified to ${f}_{{\rm{e}}}^{{\rm{SA}}}/({f}_{{\rm{e}}}^{{\rm{DA}}}{)}^{2}$, in which a geometric mean of the high- and low-energy values is used as a common collection efficiency for both electrons of the pair. The actual electron energies in the pair distributions can be read from the coincidence maps displaying the general sharings of energy between the electron pairs. After other correction detailed in later sections, percentages of double Auger decay in the samples examined in this study are collected in Table 1. The Table also includes values determined in our previous study on this subject published in ref.^3^.

**Table 1:** Percentage of double to single Auger decay in comparatively simple molecules, their respective correction factor (f_corr_), and the number of available valence electrons (N_ve_).

Molecule	f_corr_	DA [%]	N_ve_
Ne 1s	1.617	5.7 ± 1.7	
Ar 2p	1.792	9.0 ± 1.2	
**Carbon 1s**			
CH_4_		6.3^a^ ± 0.6	8
C_2_H_2_		8.2^a^ ± 0.8	10
C_2_H_4_		8.5^a^ ± 0.8	12
C_2_H_6_		8.8^a^ ± 0.8	14
CO		9.2^a^ ± 0.8	10
CO_2_		14.3^a^ ± 1.3	16
CH_3_Cl	1.74	16.5 ± 2.1	20
CF_4_	1.764	15.8 ± 2.0	32
CS_2_	1.748	22.8 ± 3.0	32
CCl_4_	1.786	29.1 ± 3.7	56
**Nitrogen 1s**			
NH_3_		8.2^a^ ± 0.8	8
N_2_		9.4^a^ ± 0.9	10
**Oxygen 1s**			
H_2_O		6.6^a^ ± 0.7	8
O_2_		7.1^a^ ± 0.7	12
CO		9.2^a^ ± 0.9	10
CO_2_		9.7^a^ ± 0.9	16
SO_2_	1.629	13.7 ± 2.0	26
**Fluorine 1s**			
CF_4_	1.55	9.6 ± 1.7	32
SF_6_	1.573	11.9 ± 2.1	56
**Sulphur 2p**			
D_2_S	1.80	8.8 ± 1.1	8
CS_2_	1.80	12.7 ± 1.6	16
C_4_H_4_S	1.80	13.2 ± 1.7	26
SO_2_	1.807	16.6 ± 2.1	18
SF_6_	1.807	23.1 ± 2.9	48
**Chlorine 2p**			
HCl	1.80	9.0 ± 1.1	8
CH_3_Cl	1.794	10.2 ± 1.3	14
CCl_4_	1.794	13.5 ± 1.7	32
SiCl_4_	1.794	14.6 ± 1.9	40
**Silicon 2p**			
SiCl_4_	1.814	18.1 ± 2.3	32

^a^Obtained in our previous study in ref.^3^.

For comparison and as a test of our method the fractions of triple ionization (DA) after hole creation in Ne and Ar were determined in the same way as for the molecules. Our new value of 5.7% DA in Ne after 1s^−1^ photoionization agrees with our previous measurement of 5.3%^3^ within the given error bars. Kanngiesser *et al*.^14^ found a value close to 6% of Ne^3+^ from 1s^−1^ photoionization which is also in line with our measurements. For Ar the value of 9.0% DA upon 2p^−1^ photoionization is in very good agreement with the values of 9.1% and 9.4% that Lablanquie *et al*.^15^ obtained from photoionization measurements involving the $2{{\rm{p}}}_{\mathrm{3/2}}^{-1}$ and $2{{\rm{p}}}_{\mathrm{1/2}}^{-1}$ core holes, respectively. Furthermore, Saito and Suzuki^16^ obtained 10% from their electron/ion coincidence measurements which is also in reasonably good agreement.

**Figure 1:**
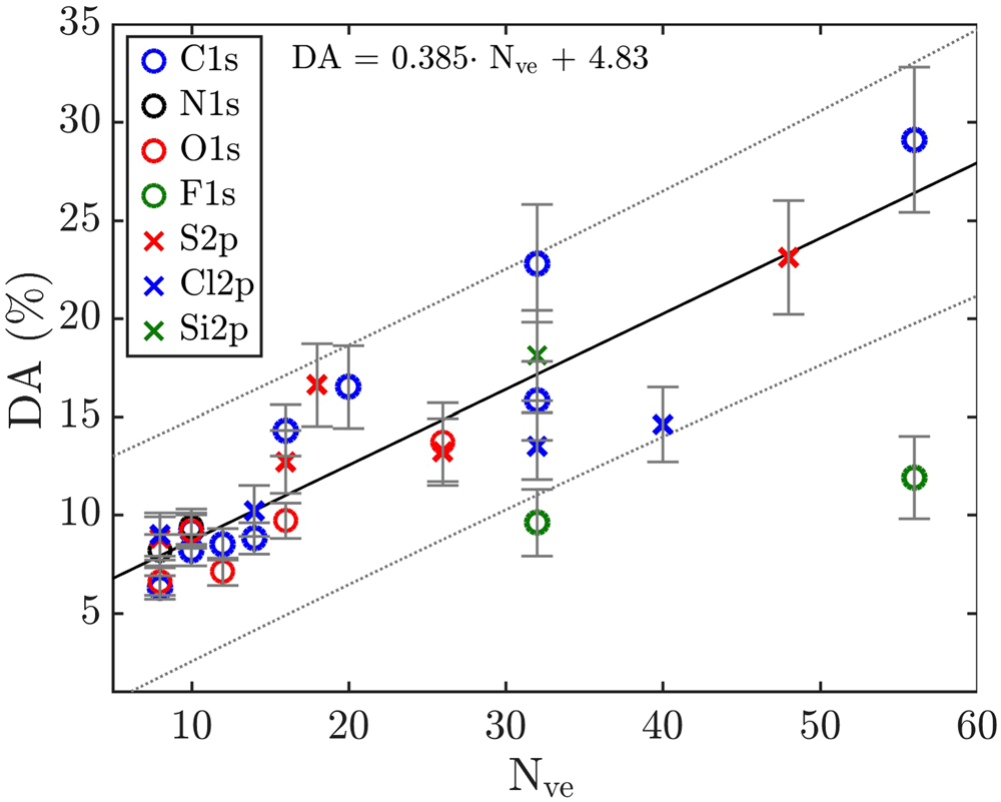
Percentage of double Auger as a function of the number of available valence electrons. A robust regression (solid line) show the trend among the molecules in Table 1, with a 95% confidence interval (dotted line) for the fit. The standard error in each data point is displayed as an error bar.

The percentages of DA for the molecules listed in Table 1 are plotted in Fig. 1 against the conventional number of valence electrons in each molecule. The reason for plotting the proportion of DA in this way was discussed in detail already in our previous work on the extent of double Auger decay^3^, and is based on a zeroth order model supported by Fermi’s golden rule. The valence electrons in each molecule are the electrons that may be involved in the Auger process, and their number is related to the density of final states in the triply-charged products. The trend noticed in our previous study^3^ for the amount of double Auger decay to increase as a function of the number of valence orbitals or equivalently valence electrons is also seen in the present larger data set. But a few of the new data points, particularly where the core hole is created in F 1s, deviate from the general trend, which indicates that the zeroth-order model is most probably too simplistic. There could be several reasons for this deviation, but a large contribution may come from the inverse energy dependence for double Auger decay discussed by Amusia *et al*.^1^. This inverse energy dependence of the fraction of double Auger is readily seen in comparison between the values for DA for Ne and Ar in Table 1. The valence shell strucure and valence electron count are exactly the same in Ne and Ar, but there is considerably more energy to be shared by the electron pair in double Auger decay upon Ne 1s ionization as against Ar 2p ionization. Amusia *et al*. discussed the theory for this inverse energy dependence within the framework of many-body perturbation theory for the case of atoms, but much of this discussion can be expected, at least approximately, to be valid also in the case of molecules. However, it is very difficult to make a quantitative estimate of the effects of this inverse energy dependence, mainly because of the continuous energy sharing between the two electrons in the double Auger process.

Other factors that might cause divergence from a linear relationship to the total number of valence electrons are the location of the core vacancy within the molecular framework, and dissociation processes induced by the core vacancy, prior to final electron emission decay. Both of these factors may modify the effective number of electrons actually available. Theoretical calculations on Auger transition rates in molecules indicate that the lifetime of a core vacancy should exhibit molecular effects^17–20^, but calculations based on different approximations may exhibit large discrepancies. For instance, calculations based on the one-centre model^17,20^ indicate a trend towards lower decay rates as electronegative ligand atoms withdraw valence electrons from the vicinity of the core vacancy, while calculations based on multi-centre models may indicate the opposite. This can be seen in calculations of Hartmann^18,19^ which seem to indicate that the lifetime of the core vacancy in CF_4_ and CCl_4_ is shorter than for CH_4_. The obvious difference between these models is that the multi-centre models also allow the valence electrons on the ligand atoms to participate in the Auger decay. Further indications that this may be the case for some systems can be seen in the work of Kay *et al*.^21^.

**Figure 2:**
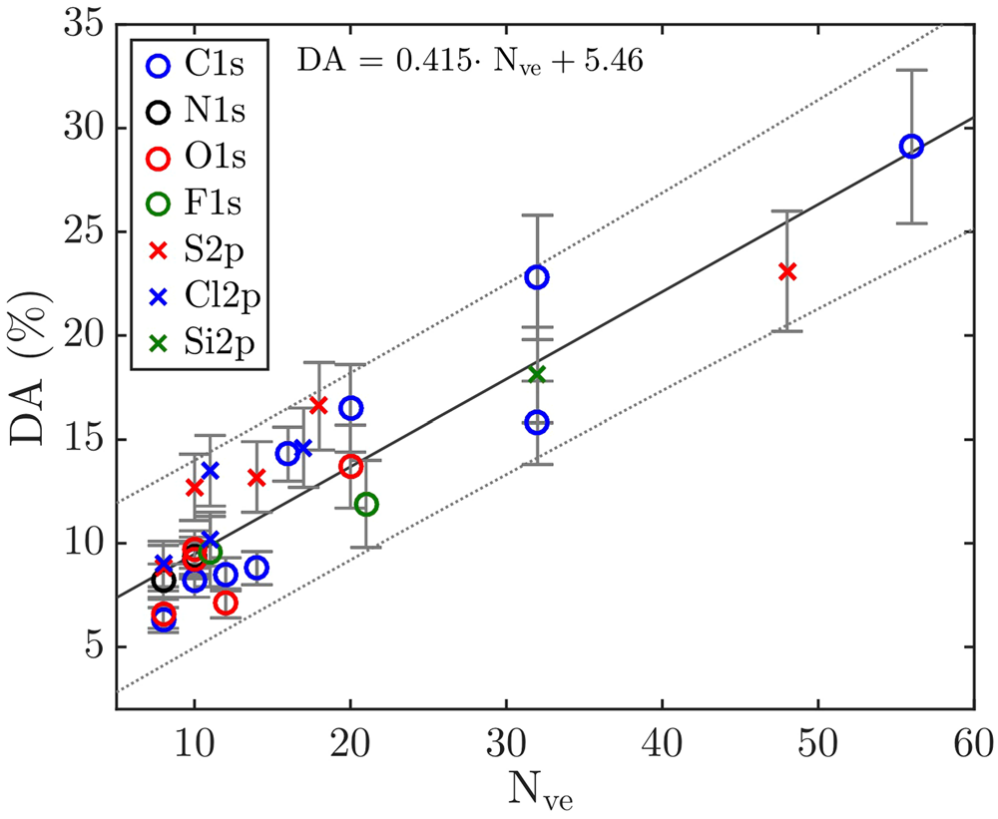
Percentage of double Auger as a function of the closest neighbouring valence electrons. The linear fit (solid line) show the trend among the molecules in Table 1, with a 95% confidence interval (dotted line) for the fit. The standard error in each data point is displayed as an error bar.

Also, a question that has to be considered in the present context is whether all valence electrons in a molecule participate in the Auger decay process or if some can be neglected, and if this could be another reason for the deviations from the linear trend observed in Fig. 1. As seen in the calculations on the Auger KVV lineshapes in molecular oxygen carried out by Wormeester *et al*.^22^, the contribution from the inter-atomic transitions in the Auger process decreases as the O-O interatomic distance is increased and may even be neglected at an O-O distance of 2 Å, while at the equilibrium O-O distance the contributions are significant. The bond distance appears to be one of the key factors, and neighbouring atoms with the shortest bond distance could be expected to contribute most to the strength of the inter-atomic Auger transition as demonstrated by Wormeester *et al*.^22^. This suggests that the number of electrons actually available for the Auger process in polyatomic molecules should primarily include those located on the atom where the core hole was created and electrons of the nearest neighbouring atom(s). This idea is explored in Fig. 2, where the fraction of DA is shown as a function of the number of valence electrons on the atom with the core hole and on closest neighbour atoms within an equilibrium distance shorter than 2 Å. This threshold for the interatomic distance to neighbouring atoms, and therefore for the number of available valence electrons, is arbitrarily chosen to be shorter than 2 Å for all of the studied molecules, which is the O-O distance where the contribution from the inter-atomic transitions in the Auger process may be neglected^22^. As can be seen, in this figure the linear trend is now more closely adhered to than in Fig. 1, and all of the previously diverging data points are now within the confidence interval of the linear trend. The fitted linear trend has the form DA = 0.415 $\cdot$ N_ve_ + 5.46, and could be used as a means to obtain a rough estimate of the proportion of DA in other molecules not mentioned in this study. The most interesting aspect of this trend for possible theoretical interpretation is the slope of the fitted line, as its extrapolation to the case where there are no valence electrons cannot have a physical meaning. Of course, such a treatment of the amount of double Auger decay is a simplification, because it does not include all decay pathways. However, even though it is a further simplification to consider only closest neighbouring valence electrons, it seems to be a good approximation. This conclusion is also supported by the work of Arion *et al*.^23^ who found that the CF_4_ molecule has (single) Auger decay pathways where the molecule can end up in ${{\rm{F}}}_{1}^{-1}$ ${{\rm{F}}}_{2}^{-1}$ final states with valence vacancies on different fluorine atoms, but where a large amount of the final states ends up with a F^−2^ configuration.

As a consequence of this model we anticipate, for instance, that in cases where an initial core vacancy is at a centre of molecular symmetry, a higher amount of double Auger decay may be produced relative to cases where the initial core vacancy is located off centre. But this linear model fitting the fraction of double Auger decay (cf. Fig. 2) is a only a simple empirical approximation and cannot be expected to provide detailed predictions. We hope that it will inspire new theoretical investigations, using thorough quantum chemical methods. It is not clear to what extent the amount of DA in molecules depends on the different factors discussed, like the location of the core vacancy, the electron density, the dissociation dynamics, and the total available energy for the double Auger process. One further observation that can be made from Figs 1 and 2 is that the extent of double Auger decay after 1s and 2p core hole formation events follow the same linear trend, which may indicate that the hole identity is not a crucial distinction for the proportion of double Auger decay. The linear fit model does not take into account the probable energy dependence of the double Auger decay, but it does give a rough estimate of the amount of DA decay depending on the location of the initial core vacancy in molecules of different sizes. To get a better understanding of the contributing factors and to what extent they contribute to the relative proportions of single and double Auger decay in molecular systems, further theoretical and experimental studies will have to be carried out.

## Conclusions

We have extended measurements of the ratio of double to single Auger decay after 1s or 2p core hole formation in molecules containing C, O, F, Si, S and Cl atoms, using electron-electron coincidences to measure the relative abundance of single electrons and electron pairs emitted after initial core-hole creation. Single and double Auger spectra were recorded for each molecule and after correction for the different collection efficiencies at different energies, yielded the relative percentages of double and single Auger decays. The fraction of double Auger decay after creation of a 1s or a 2p core hole is substantial, surpassing 20% in some of the heavier molecules, which is much more than has typically been assumed in the past. The clearest trend among the molecules is that the larger molecules have a larger proportion of double Auger decay, and the proportion is observed to follow a linear trend correlated to the number of available valence electrons on the atom with the core hole and on closest neighbouring atoms, where the interatomic distance is assumed to play a large role in the observed proportion. No significant difference in the amount of double Auger decay after 1s or 2p core hole formation can be seen, and the two cases follow the same linear trend, which may indicate that the hole identity is not a crucial factor for the proportion of double Auger decay. Further experimental and theoretical investigations of the proportion of double Auger decay would be highly desirable and would be highly relevant, as is the present work, to studies of dissociative ionization after site-specific core hole formation, particularly where only ions are detected.

## Methods

### Experimental

As in our previous work^3^ the experiments were done on beam line UE52 SGM of the synchrotron radiation facility BESSY-II in Berlin, operating in single-bunch mode, using a magnetic bottle time-of-flight electron spectrometer which was described in detail before (see ref.^24^). Here we mention only the essential characteristics of the apparatus and arrangements specific to the present work. The magnetic bottle is a 2 m instrument whose magnetic field arrangement consists of a conically shaped permanent magnet close to the light-matter interaction region and a solenoid wound around the flight tube. At the end of this flight tube essentially all electrons emitted into a solid angle of 4*π* impinge upon a microchannel plate (MCP) detector. Electron flight times measured relative to the synchrotron radiation pulses are converted by a comparatively simple formula^24^ to electron kinetic energies. Calibration of the flight-time to energy conversion is based on well-known Auger and photoelectron lines of Kr, Ar, and Ne^25–27^. Energy resolution ranges from about 50 meV for 1 eV electrons to about 10 eV for electrons of 600 eV. To maintain constant collection efficiency for the electrons throughout the measurements, all experimental parameters including the positioning of the permanent magnet relative to the solenoid and the gas needle as well as the strength of the solenoid field and the electric potentials applied to the source region were kept constant after optimization. Because the detection efficiency depends on the gain of the MCP detector, determined by the voltages applied, and on the settings of the gain and discriminator levels in the electronics these were also kept constant. The rate of accidental coincidences was reduced to a negligible level by keeping the total electron count rate at less than 800 Hz, far lower than both the light pulse rate of 1.25 MHz and the more relevant inverse of the total electron flight time range of 5 *μ*s, 200 kHz.

### Calibration of the electron collection and detection efficiency

**Figure 3:**
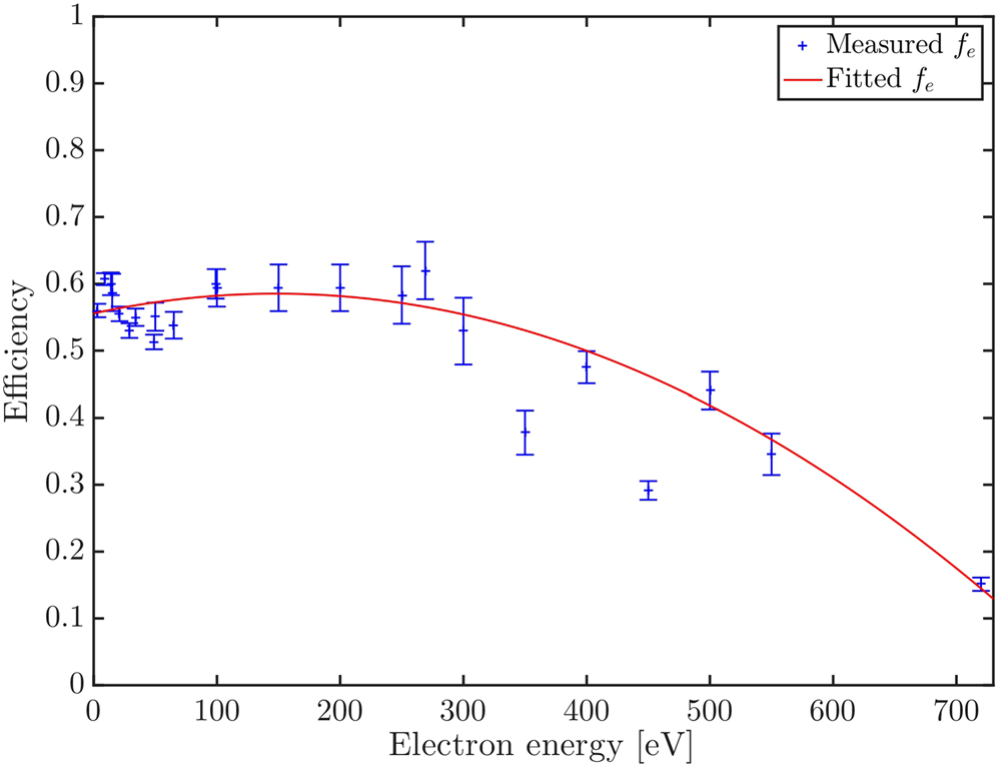
Combined collection-detection efficiency of the magnetic bottle electron spectrometer used in the present study as a function of electron kinetic energy. Error bars display the standard deviation for the efficiency in each data point.

As in our previous work^3^, the combined collection-detection efficiency (written here again as collection efficiency) was determined over the full energy range by single Auger decay measurements on neon, argon, and krypton. The new experiments used longer run times and so achieved better statistics and precision than the earlier work. Absolute collection efficiency values were extracted based on the expressions of the total detected coincidences (C_SA_) of one Auger electron and one photoelectron

$${{\rm{C}}}_{{\rm{SA}}}={{\rm{N}}}_{{\rm{SA}}}\,{f}_{{\rm{e}}}^{2},$$

and the detection of only one Auger electron (A_SA_) as

$${{\rm{A}}}_{{\rm{SA}}}={{\rm{N}}}_{{\rm{SA}}}\,{f}_{{\rm{e}}}\mathrm{(1}-{f}_{{\rm{e}}}),$$

where N_SA_ is the true number of single Auger events in the source region and ${f}_{{\rm{e}}}$ is the collection efficiency. The collection efficiency may then be determined from the ratio ${f}_{{\rm{e}}}={{\rm{C}}}_{{\rm{SA}}}/({{\rm{A}}}_{{\rm{SA}}}+{{\rm{C}}}_{{\rm{SA}}})$, in conditions where the collection efficiency can be assumed to be the same for photo- and Auger electrons. This condition is fulfilled for ionization of Kr at 110 eV photon energy, for instance, as the photoelectron energies are near 15 eV while the Auger electron energies are between 20 and 60 eV. Earlier experiments^3^ have shown that collection efficiencies do not vary appreciably in this energy range. Although triple ionization of Kr also occurs at 110 eV, we eliminate the resulting interference by selecting resolved Auger electrons and subtracting continuous background contributions. Collection efficiencies covering a wider range of energies were determined from single Auger decay measurements on Ar at photon energies from the Ar 2p^−1^ thresholds (249.7 eV) up to 520 eV and on Ne from the 1s threshold (870.2 eV) up to 1420 eV. In this range a different calibration procedure is needed, because collection efficiencies for the photoelectron and Auger electron will differ since the energies are very different, and spectral overlap may affect the data. The rather low resolution (wide bandwidth) means that Ne Auger electrons of about 750–810 eV energy, are not fully separated from valence photoelectrons on the high energy side, nor from double Auger contributions on the low energy side. In addition, there is an inextricable overlap with Auger electrons from satellite states. All of these overlaps increase the measured intensity of the Auger signal relative to a pure single Auger signal, in both the Ne and the Ar cases. However, as an approximation these contributions to the Auger signals can be considered to be unvarying over the energy ranges considered here. Cross-sections for valence single photoionization and direct multiple photoionization are much lower than for core photoionization, and the relative contribution from double Auger is definitely constant, as it and the main Auger signal come from formation of the same core hole. Furthermore, from the cases where the satellite states could be resolved we know that the contributions from satellites are comparatively small and are relatively constant (±5% units). Based on these assumptions the total detected coincidence and Auger electrons can be written as

$${{\rm{C}}}_{{\rm{SA}}}={{\rm{N}}}_{{\rm{SA}}}\,{f}_{{\rm{e}}}^{{\rm{A}}}{f}_{{\rm{e}}}^{{\rm{P}}}$$

$${{\rm{A}}}_{{\rm{SA}}}={{\rm{N}}}_{{\rm{SA}}}\,{f}_{{\rm{e}}}^{{\rm{A}}}\mathrm{(1}-{f}_{{\rm{e}}}^{{\rm{P}}}),$$

where the collection efficiencies are written as ${f}_{{\rm{e}}}^{{\rm{A}}}$ and ${f}_{{\rm{e}}}^{{\rm{P}}}$ with superscripts to identify the Auger electrons and photoelectrons. The apparent collection efficiency ${f}_{e}^{^{\prime} }=k{{\rm{C}}}_{{\rm{SA}}}/({{\rm{A}}}_{{\rm{SA}}}+{{\rm{C}}}_{{\rm{SA}}})$ is reduced, because of overlap effects and is scaled by a factor *k*, whose values for Ar and Ne are estimated by comparing efficiencies determined from measurements on Kr with similar normalised measurements on the other two gases. The results for the collection efficiencies obtained are shown in Fig. 3. The efficiency, which is fitted with a robust regression to the data points, is around 58% from zero up to about 300 eV photon energy and goes down slowly to 15% at 720 eV. Despite the improved statistics and tighter error bars compared to our previous work^3^, collection efficiencies are still the main source of uncertainty on the proportions of DA and SA decay obtained in this study.

### Treatment of false coincidences

Because of the low rate of ionization events used in the present work, the rate of truly accidental coincidences originating from more than one ionization event per light pulse is negligible. But there are many real but unwanted coincidences, whose rate must be minimized in the experiments or allowed for in the data treatment. The origin of the majority of these unwanted coincidences is the release of secondary electrons from impact of stray photons, photo- or Auger electrons on metal surfaces, primarily the conical magnet and the gas needle. The largest contribution is made by fast electrons from single Auger decay of the target molecules, whose impact on a surface can generate one or more secondary electrons, leaving the associated photoelectron to be detected in the usual way. This will produce unwanted double, triple or higher order coincidences. The secondary electrons generated by impact are of relatively low energy and have a continuous distribution; they therefore generate false signals at high ionization energies. Their effect can be greatly reduced by selecting events with total electron energy sum corresponding to the correct range of double or triple ionization energy. A second significant contribution can come from primary photoelectrons hitting a surface and generating secondaries while the Auger electrons are detected normally. In this case the summed electron energy is quite likely to be in an acceptable energy range for double or triple ionization, so to deal with these false coincidence signals we select Auger events only in coincidence with the desired photoelectron. A different potential source of error is electron energy loss by inelastic collisions with residual gas in the flight tube or with surfaces. Electrons which have lost energy may still be detected, and some are seen as weak tails extending over a few eV on the low energy side of sharp ionization peaks. The magnitude of the effect is hard to estimate, but where the tails can be clearly seen their integrated intensity is of the order of 2–4% of the original peak. For the sharp photoelectron peaks any tails are rejected in the analysis, while for molecular Auger electrons, which have broad energy spreads, the major parts of any tails will be automatically included correctly in the measured peak areas.

## Data Availability

The datasets generated during and/or analysed during the current study are available from the corresponding author on reasonable request.
